# Noninvasive Prediction of *TP53* Gene Status and *ATRX* Gene Status in *IDH*-Mutant Glioma Using Multimodal MRI: Incorporating Morphological, Spectroscopic, Diffusion, and Perfusion Imaging

**DOI:** 10.3390/diagnostics16142174

**Published:** 2026-07-12

**Authors:** Sixuan Chen, Zhengyang Zhu, Huiquan Yang, Meiping Ye, Yang Song, Chuanshuai Tian, Fengnan Niu, Zhengge Wang, Xin Li, Xin Zhang, Bing Zhang

**Affiliations:** 1 Department of Radiology, Nanjing Drum Tower Hospital, Affiliated Hospital of Medical School, Nanjing University, Nanjing 210008, China; 2 Institute of Medical Imaging and Artificial Intelligence, Nanjing University, Nanjing 210008, China; 3 Medical Imaging Center, Department of Radiology, Nanjing Drum Tower Hospital, Affiliated Hospital of Medical School, Nanjing University, Nanjing 210008, China

**Keywords:** glioma, isocitrate dehydrogenase-mutant, magnetic resonance imaging, multimodal, genetics

## Abstract

**Background/Objectives**: Noninvasive determination of glioma molecular profiles is clinically crucial for assessing therapeutic efficacy and predicting disease outcomes. This study aimed to evaluate the potential of morphological magnetic resonance imaging (MRI), diffusion-weighted imaging (DWI), magnetic resonance spectroscopy (MRS), and dynamic contrast-enhanced perfusion-weighted imaging (DCE-PWI) in predicting *TP53* gene status and X-linked alpha-thalassemia intellectual disability syndrome (*ATRX*) gene status in isocitrate dehydrogenase (*IDH*)-mutant gliomas. **Methods**: A retrospective analysis was performed on 106 *IDH*-mutant glioma patients using morphological MRI, DWI, MRS, and DCE-PWI data. Statistical comparisons of imaging parameters across molecular status groups were conducted, and logistic regression models were developed to predict molecular status, with diagnostic performance evaluated by receiver operating characteristic (ROC) curve analysis. Five-fold stratified cross-validation with 1000 bootstrap resamples was employed to assess model generalizability **Results**: Among 106 *IDH*-mutant gliomas, the *TP53*-mutant group showed a greater proportion of tumors with >33% enhancement (*p* = 0.018), higher Cho/Cr (*p* < 0.001), and higher Cho/NAA (*p* = 0.005) than the *TP53*-wildtype group. Multivariable analysis demonstrated that the Cho/Cr ratio was an independent predictor of *TP53* mutation in *IDH*-mutant gliomas (odds ratio [OR] = 2.037, *p* = 0.021), with the model achieving an apparent AUC of 0.741. DCE-PWI parameters showed no significant differences across molecular subgroups. Ve was significantly elevated in *ATRX*-mutant tumors (median 57.16 vs. 30.63, *p* = 0.029). Ktrans, Kep, Vp, and iAUC showed no significant differences between groups (all *p* > 0.05). Furthermore, multivariable analysis showed that ADC values (OR = 1.005, *p* = 0.017) and the Cho/NAA ratio (OR = 3.073, *p* = 0.023) emerged as independent predictors of *ATRX* mutation, with the model achieving an apparent AUC of 0.863. Five-fold cross-validation demonstrated that the Cho/Cr model for TP*53* prediction achieved a mean AUC of 0.717 ± 0.043 (Bootstrap 95% CI: 0.616–0.814), and the ADC + ChoNAA model for *ATRX* prediction achieved 0.865 ± 0.124 (95% CI: 0.780–0.953). All predictors remained significant across all five folds. Pooled confusion matrices yielded sensitivities of 0.623 and 0.757, specificities of 0.696 and 0.909, and accuracies of 0.654 and 0.840, respectively. **Conclusions**: Multimodal MRI techniques (morphological MRI, DWI, MRS, and DCE-PWI) can help predict *TP53* and *ATRX* status without surgery. Higher Cho/Cr and Cho/NAA ratios were independently associated with *TP53* mutation, whereas lower ADC and higher Cho/NAA independently predicted *ATRX* mutation. These findings suggest that a focused imaging protocol may be sufficient for preoperative molecular profiling in this tumor type.

## 1. Introduction

According to the fifth edition of the classification of central nervous system (CNS) tumors by the World Health Organization (WHO), diffuse astrocytic tumors with *IDH* mutation are now classified as *IDH*-mutant glioma [1]. This reclassification is prompted by the notable variances in the genetic and epigenetic characteristics between *IDH*-mutant glioma and *IDH*-wild-type glioma. Additionally, it is important to note that the clinical outcomes of *IDH*-mutant gliomas can differ widely depending on coexisting molecular alterations. Astrocytoma, *IDH*-mutant, is characterized by concurrent *ATRX* inactivation and *TP53* mutation, along with the absence of 1p/19q codeletion [2,3]. This new understanding of glioma subtypes has important implications for treatment and prognosis [4].

*ATRX* gene mutation has emerged as a significant biomarker with strong associations to glioma subtype and likelihood of recurrence [5]. In diagnostic practice, loss of *ATRX* nuclear expression is a definitive marker used to differentiate *IDH*-mutant astrocytomas from oligodendrogliomas, directly informing the integrated WHO diagnosis [6]. Studies have suggested that *ATRX* gene mutations may affect the response to certain treatments. This molecular feature is independently associated with distinct prognosis and patterns of treatment failure [7,8].

*TP53* gene status is a key molecular marker in the classification of *IDH*-mutant gliomas, aiding in the distinction between astrocytomas and oligodendrogliomas [9,10]. Wild-type *P53* usually inhibits the generation of gliomas and its activity can be harnessed through targeted therapies or chemotherapy to control disease progression. *P53* protein often exhibits gain-of-function or dominant-negative effects, promoting oncogenesis by inhibiting apoptosis, enhancing tumor malignancy, conferring resistance to targeted therapies, and reducing treatment efficacy [11,12,13].

Traditional glioma genotyping relies on immunohistochemistry or gene sequencing. These methods require invasive biopsy or surgical resection for pathological results, which not only poses risks and irreversible trauma to patients, but also has expensive testing costs and long waiting times [14]. Furthermore, while immunohistochemical interpretation is guided by standardized scoring protocols, diagnostic accuracy can still be influenced by variability in tissue sampling and inherent technical limitations.

While conventional morphological MRI features such as cystic changes, necrosis, peritumoral edema, and contrast enhancement have been explored for predicting the molecular profiles and survival in glioma patients [15], their diagnostic specificity is often limited. More robust and validated radiogenomic predictors, such as the T2/FLAIR mismatch sign [16] and the presence of calcifications, have emerged as stronger non-invasive indicators of specific molecular subtypes and clinical outcomes. DWI is a non-invasive imaging technique that can provide valuable insights into the microscopic properties of tissues by evaluating the random movement of water molecules, known as Brownian motion. In brain tumors, restricted diffusion, reflected by a low apparent diffusion coefficient (ADC), typically indicates high cellularity, which is a hallmark of aggressive tumor biology. Clinically, markedly low ADC values are associated with high-grade gliomas and may correlate with a poorer response to therapy, as often observed in glioblastoma [17,18,19]. DCE-PWI is a non-invasive imaging technique that uses perfusion-weighted MRI to assess the density, integrity, and permeability of tissue blood vessels, offering supplemental insights beyond those obtained from standard MRI. Previous studies have utilized perfusion MRI to examine genetic mutations in glioma patients. These findings are clinically relevant, as high perfusion is frequently linked to aggressive tumor behavior, higher histological grade, and adverse outcomes, serving as an imaging biomarker for treatment resistance [20,21,22]. In vivo proton magnetic resonance spectroscopy (^1^H-MRS) provides detailed chemical profiling of specific brain regions and has been utilized to link biochemical alterations with various central nervous system pathologies. In neuro-oncology, an elevated choline (Cho) peak indicates increased cell membrane turnover and proliferation, a key feature of active tumor growth. Metabolite ratios, such as an elevated Cho/N-acetylaspartate (NAA) ratio, are valuable for tumor grading and differentiation from non-neoplastic lesions [23]. MRS aids in differentiating tumor recurrence from post-treatment effects such as radiation necrosis. By integrating metabolic profiles with structural MRI, MRS enhances diagnostic accuracy, supports personalized treatment planning, and contributes to the advanced molecular characterization of gliomas in the era of precision neuro-oncology [24,25].

To our knowledge, remarkably few investigations have integrated morphological MRI with MRS, DWI, and perfusion techniques for concurrent evaluation of *ATRX* and *TP53* gene status in *IDH*-mutant gliomas.

Our research assessed the diagnostic accuracy of multiparametric MRI including morphological MRI, DWI, MRS, and DCE-PWI in categorizing *IDH*-mutant glioma based on molecular indicators including *ATRX* mutation status and *TP53* gene status in *IDH* mutant gliomas.

## 2. Materials and Methods

### 2.1. Patient Enrollment

**Figure 1:**
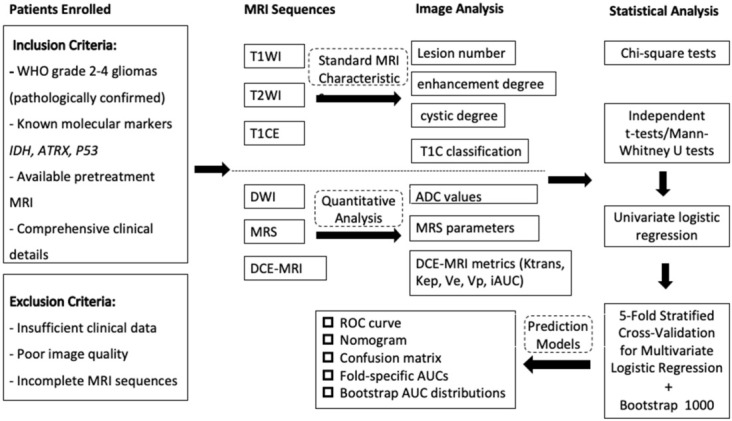
The overview of the experimental design. Patients were enrolled according to the above-mentioned criteria. This retrospective study analyzed *IDH*-mutant glioma patients using multi-parametric MRI data, including DWI, MRS, and DCE-PWI. Categorical MRI characteristics were compared using chi-square tests or Fisher’s exact test as appropriate. Continuous variables, including apparent diffusion coefficient (ADC) values, MRS parameters (NAA/Cr, Cho/Cr, Cho/NAA ratios), and DCE-PWI parameters (Ktrans, Kep, Ve, Vp, iAUC), were analyzed using Student’s *t*-test or Mann–Whitney U test to assess their association with molecular marker status. Logistic regression models were developed to predict molecular subtypes, with diagnostic performance evaluated through ROC curve analysis and nomogram. For internal validation, a 5-fold stratified cross-validation with 1000 bootstrap resamples was performed to assess model generalizability. In each fold, 4/5 of the data served as training for model fitting and cutoff determination, while 1/5 served as testing for independent performance evaluation. The mean AUC ± standard deviation and 95% confidence intervals were reported.

Patients were retrospectively recruited from the Affiliated Drum Tower Hospital of Nanjing University Medical School between July 2019 and July 2025. The Institutional Review Board approved the study and waived the requirement for informed consent. A total of 140 patients were initially identified. The inclusion criteria were as follows: (1) pathologically confirmed WHO grade 2–4 gliomas; (2) known *IDH* mutation status, *ATRX* mutation status, and *TP53* gene status; (3) availability of pretreatment morphological MRI, MRS, DWI, and DCE-PWI; and (4) comprehensive clinical details, including age, sex, and histological grade. Molecular analyses were performed for all enrolled patients; however, not all patients were tested for every molecular marker. After excluding 34 patients due to insufficient clinical data (*n* = 14), poor image quality (*n* = 9), or lack of complete MRI sequences (*n* = 11), 106 patients were included in the final analysis. The workflow of patient enrollment is shown in Figure S1. *TP53* immunohistochemistry was performed routinely and was available for all 106 patients. *ATRX* immunohistochemistry was not part of the initial standard pathology workflow at our institution, and was added to the standard pathology workflow later in the study period, so *ATRX* status was available for 81 patients enrolled after January 2021. The other 25 patients had unknown *ATRX* status and were not included in *ATRX*-specific analyses. The overview of the experimental design is shown in Figure 1.

### 2.2. MRI Protocol

All patients underwent preoperative MRI examinations using two 3T scanners: a United Imaging uMR790 scanner (United Imaging Healthcare, Shanghai, China; *n* = 58) and a Philips Ingenia CX scanner (Philips Medical Systems, Best, The Netherlands; *n* = 54). The imaging protocol for both scanners included the following sequences: T1-weighted imaging (T1WI), T2-weighted imaging (T2WI), diffusion-weighted imaging (DWI), contrast-enhanced T1-weighted imaging (CE-T1WI), apparent diffusion coefficient (ADC) maps, and dynamic contrast-enhanced MRI (DCE-MRI). Details of the scanning parameters are shown in the Supplementary Materials (Table S1).

### 2.3. Image Analysis

**Figure 2:**
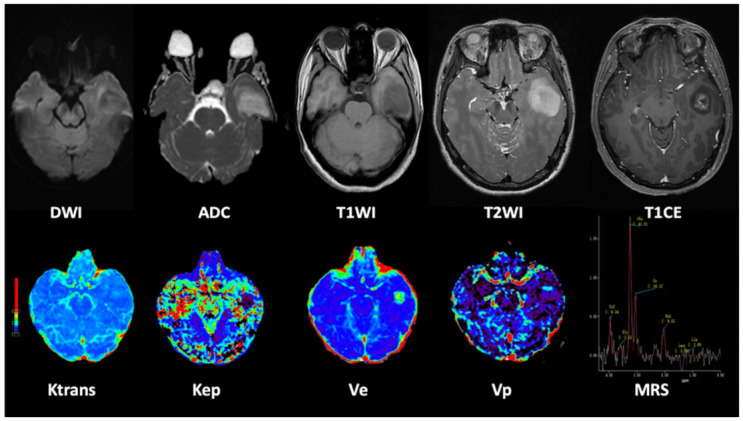
Representative multimodal MRI images (morphological, spectroscopic, diffusion, and perfusion) from a 41-year-old female with a pathological diagnosis of anaplastic glioma, WHO grade 3. This lesion is well-defined, exhibits heterogeneous enhancement, demonstrates relatively high ADC values, shows relatively low perfusion, and has a Cho/NAA ratio of approximately 2.8. Molecular testing demonstrated an *IDH*1 mutation, retained *ATRX* expression, and a *TP53* mutation. The color scale represents the parameter values for each pixel, with red indicating higher values and blue indicating lower values.

**Figure 3:**
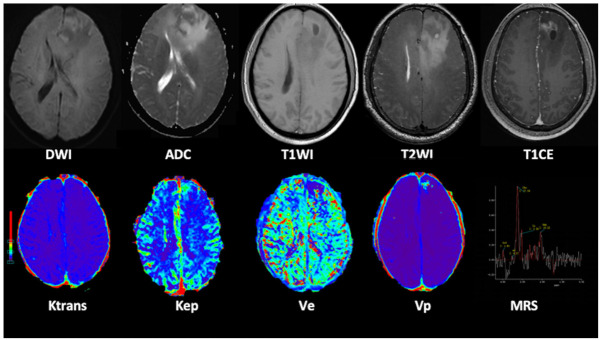
Representative multimodal MRI images (morphological, spectroscopic, diffusion, and perfusion) from a 51-year-old male with a pathological diagnosis of diffuse glioma, WHO grade 2. This lesion is ill-defined, exhibits heterogeneous enhancement, demonstrates relatively low ADC values, shows relatively low perfusion, and has a Cho/NAA ratio of approximately 3.2. Molecular testing demonstrated an *IDH*1 mutation, retained *ATRX* expression, and wild-type *TP53*. The color scale represents the parameter values for each pixel, with red indicating higher values and blue indicating lower values.

The standard MRI characteristics, such as the number of lesions, degree of enhancement, cystic degree, and TIC classification, were evaluated using T1WI, T2WI, and CE-T1WI images. The degree of cystic change and contrast enhancement within each tumor were assessed qualitatively by two independent neuroradiologists. Both features were visually estimated as a percentage of the total tumor volume and subsequently categorized as follows: cystic degree was classified as either ≤25% or >25%, representing the estimated proportion of cystic/necrotic components; enhancement degree was classified as either ≤33% or >33%, representing the estimated proportion of contrast-enhancing tumor tissue. Additionally, quantitative analysis of ADC values was performed. Standard MRI features, including lesion number, enhancement degree TIC classification and cystic degree, were assessed on T1WI, T2WI and CE-T1WI images. TICs were classified into three types according to a previous report [26]. Quantitative analyses of ADC values, MRS parameters, and DCE-MRI metrics were performed using United Imaging and Philips post-processing workstations. Regions of interest (ROIs) were manually delineated on a single representative axial slice showing the maximal tumor cross-sectional area within areas of maximal contrast enhancement in solid tumor components, carefully avoiding regions of hemorrhage, cystic degeneration, necrosis, and major vasculature. For non-enhancing tumors, ROIs were positioned within the hyperintense region on T2WI near the central area of the tumor. A circular region of interest (ROI) measuring 10–15 mm^2^ was applied to the ADC maps and all DCE parameter maps. Representative ROI placements were presented in Figure S2. The derived quantitative parameters included the transfer constant from plasma into the extracellular extravascular space (Ktrans), the rate constant between the extracellular extravascular space and plasma (Kep), the volume fraction of the extracellular extravascular space (Ve), the volume fraction of the blood plasma (Vp), and the initial area under the concentration–time curve (iAUC). Ktrans represents the transfer constant of contrast agent from blood plasma into the extravascular extracellular space (EES), quantifying the permeability-surface area product per unit mass of tissue. Kep denotes the rate constant describing contrast agent efflux from EES back to the vascular compartment. Ve is the EES volume per unit tissue volume. Vp represents the plasma volume per unit volume of tissue, while iAUC refers to the initial area under the concentration–time curve within the first 60 s after contrast agent arrival. Details of DCE post-processing, including AIF determination and quality control, are provided in the Supplementary Materials. All MRI images were analyzed independently by two experienced neuroradiologists, who were blinded to histopathological results. Interobserver agreement for the quantitative parameters was assessed using the intraclass correlation coefficient (ICC) with absolute agreement. Representative cases were presented in Figure 2 and Figure 3.

Histopathological data were obtained from pathology reports. Statistical analysis was performed using R software (version 4.6.0; R Core Team, 2026). Categorical variables were analyzed using chi-square or Fisher’s exact tests, as appropriate. The normality of continuous variables was assessed using the Shapiro–Wilk test. Normally distributed data were expressed as the mean ± standard deviation and compared using independent t-tests; non-normally distributed data were expressed as median (interquartile range) and compared using the Mann–Whitney U test. All clinical features and imaging parameters were selected in univariable logistic regression. All variables that exhibited statistically significant associations (*p* < 0.05) in univariable analyses were subsequently incorporated into the multivariable regression models. The presence of multicollinearity among the variables was evaluated using the variance inflation factor (VIF). The ROC curves were derived from multivariable logistic regression models using continuous predictors, with gene status as the binary outcome. The models’ discriminative ability was assessed using the area under the ROC curve, with an apparent area under the curve (AUC) reported for the full-cohort models. In addition, sensitivity and specificity were calculated at the optimal cutoff determined by the Youden index. To evaluate model generalizability and prevent overfitting, a 5-fold stratified cross-validation with 1000 bootstrap resamples was performed for internal validation. In each fold, 4/5 of the data served as the training set for model fitting and cutoff determination (Youden index), while 1/5 served as the test set for independent performance evaluation. The mean AUC ± SD across the five folds and bootstrap 95% CIs were reported. Performance metrics, including sensitivity, specificity, accuracy, F1-score, and confusion matrices, were calculated for the validated models. The predictive accuracy of the models for *ATRX* mutation and *TP53* gene status was assessed using ROC curve analysis.

## 3. Results

The agreement in assessment of qualitative imaging parameters between the two neuroradiologists was excellent, with interrater reliability values ranging from 0.85 to 0.91 (Supplementary Table S2).

### 3.1. Comparative Analysis of Clinical Characteristics and MRI Parameters of TP53 and ATRX Status in IDH-Mutant Gliomas

Clinical data, conventional magnetic resonance imaging characteristics, and advanced MRI biomarkers of *IDH*-mutant glioma patients grouped by molecular classification are displayed in Table 1 and Table 2. Age and sex did not significantly differ across groups stratified by *TP53* gene and *ATRX* gene status.

The degree of enhancement, Cho/NAA ratio, and Cho/Cr ratio were significantly different (*p* < 0.05) between *TP53* mutant and wild-type groups. Both the Cho/Cr and Cho/NAA ratios were significantly higher in the *TP53*-mutant group. The proportion of tumors with enhancement exceeding 33% was also higher in the *TP53*-mutant group. No statistically significant differences were found in the remaining clinical or imaging characteristics.

However, *ATRX*-mutant tumors showed lower Cho/NAA ratio (*p* = 0.037), lower ADC values (*p* = 0.012), and higher Ve (*p* = 0.029) higher in the *ATRX* mutation tumors. No statistically significant differences were found in the remaining clinical or imaging characteristics.

### 3.2. Logistic Regression Analysis for TP53 Gene Status Prediction

**Figure 4:**
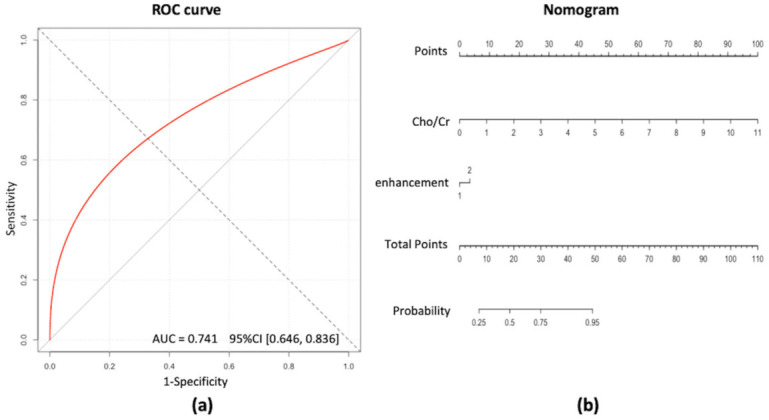
Receiver operating characteristic (ROC) curve and nomogram of the multivariable logistic regression model for predicting *TP53* status in *IDH*-mutant gliomas. (**a**) ROC curve showing the diagnostic performance of the multivariable model, including Cho/Cr ratio as the independent predictor. The apparent area under the curve (AUC) was 0.741, with a sensitivity of 72.0% and a specificity of 82.0%. The red solid line represents the ROC curve, and the gray dashed diagonal line indicates the reference line of no discrimination (AUC = 0.5). (**b**) Nomogram for predicting *TP53* mutation status based on the multivariable logistic regression model. Points are assigned to the predictor variable (Cho/Cr ratio) based on its value, and the total points correspond to the predicted probability of *TP53* mutation.

The findings from logistic regression analyses in predicting the *TP53* gene status in *IDH*-mutant gliomas are presented in Table 3. Univariable analysis revealed that a higher degree of enhancement (odds ratio [OR] = 2.612, *p* = 0.02), Kep (OR = 1.000, *p* = 0.049), Cho/Cr ratio (OR = 2.582, *p* = 0.001), and Cho/NAA ratio (OR = 1.229, *p* = 0.006) were significant predictors. In the multivariable analysis, the Cho/Cr ratio remained an independent predictor. The ROC curve and nomogram for the multivariable model are shown in Figure 4. The corresponding model exhibited an apparent AUC of 0.741. The model achieved a sensitivity of 72.0% and a specificity of 82.0%.

### 3.3. Logistic Regression Analysis for ATRX Gene Status Prediction

**Figure 5:**
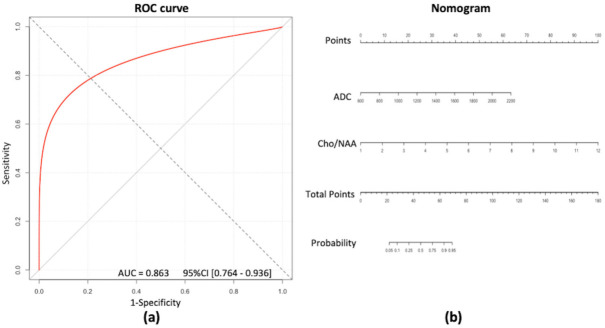
Receiver operating characteristic (ROC) curve and nomogram of the multivariable logistic regression model for predicting *ATRX* mutation status in *IDH*-mutant gliomas. (**a**) ROC curve showing the diagnostic performance of the multivariable model, including ADC values and Cho/NAA ratio as independent predictors. The apparent area under the curve (AUC) was 0.863, with a sensitivity of 81.0% and a specificity of 88.0%. The red solid line represents the ROC curve, and the gray dashed diagonal line indicates the reference line of no discrimination (AUC = 0.5). (**b**) Nomogram for predicting *ATRX* mutation status based on the multivariable logistic regression model. Points are assigned to each predictor variable (ADC and Cho/NAA ratio) based on their values, and the total points correspond to the predicted probability of *ATRX* mutation.

The findings from logistic regression analyses for predicting *ATRX* mutation status in the *IDH*-mutant gliomas are presented in Table 4. Among the quantitative MRI parameters, the Cho/NAA ratio, NAA/Cr ratio, Cho/Cr ratio, and ADC values differed significantly between *ATRX*-mutant and wild-type groups (*p* < 0.05). Univariable analysis identified higher ADC mean values (OR = 1.005, *p* = 0.017) and a higher Cho/NAA ratio (OR = 3.073, *p* = 0.023) as significant predictors. In the multivariable analysis, the corresponding model achieved an apparent AUC of 0.863. The model achieved a sensitivity of 81.0% and a specificity of 88.0%. The ROC curve and nomogram for the multivariable model are shown in Figure 5.

### 3.4. Internal Validation

**Figure 6:**
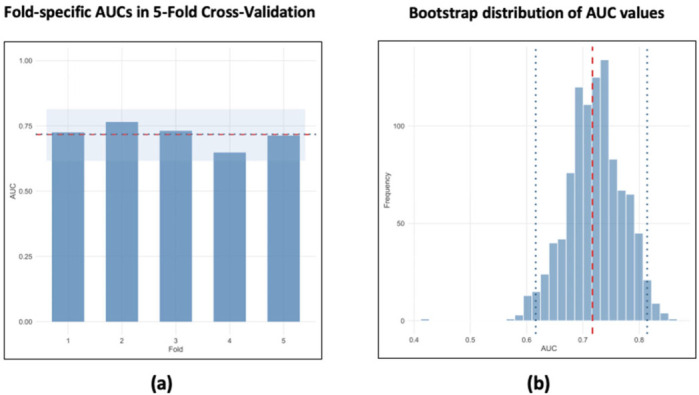
Cross-validation and bootstrap validation of the Cho/Cr model for predicting *P53* mutation. (**a**) Fold-specific AUCs in 5-fold cross-validation. Bars represent fold-specific AUCs (range: 0.648–0.765). Red dashed line: mean AUC (0.717); blue dotted line: apparent AUC (0.718); shaded area: Bootstrap 95% CI (0.616–0.814). (**b**) Bootstrap distribution of AUC values (1000 resamples). Histogram shows the frequency distribution of AUC values. Red dashed line: mean AUC (0.717); blue dotted lines: 95% CI bounds (0.616 and 0.814). Approximately normal distribution confirms AUC estimate stability.

**Figure 7:**
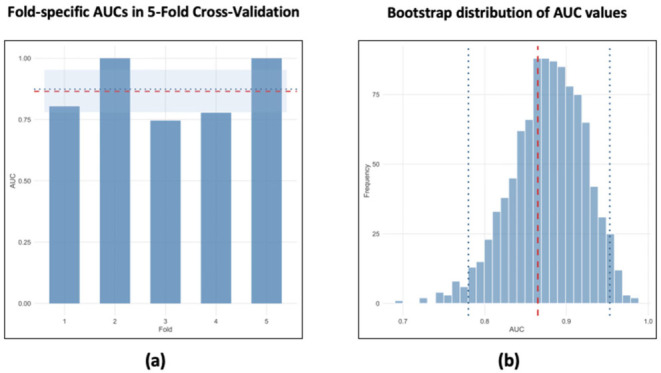
Cross-validation and bootstrap validation of the ADC + Cho/NAA model for predicting *ATRX* mutation. (**a**) Fold-specific AUCs in 5-fold cross-validation. Bars represent fold-specific AUCs (range: 0.746–1.000). Red dashed line: mean AUC (0.865); blue dotted line: apparent AUC (0.874); shaded area: Bootstrap 95% CI (0.780–0.953). (**b**) Bootstrap distribution of AUC values (1000 resamples). Histogram shows the frequency distribution of AUC values. Red dashed line: mean AUC (0.865); blue dotted lines: 95% CI bounds (0.780 and 0.953). Approximately normal distribution confirms AUC estimate stability.

Five-fold cross-validation confirmed the generalizability of both models. The Cho/Cr model for *TP53* prediction achieved a mean AUC of 0.717 ± 0.043 (Bootstrap 95% CI: 0.616–0.814). The pooled confusion matrix showed 38 true positives, 32 true negatives, 14 false positives, and 23 false negatives, yielding a sensitivity of 0.623, specificity of 0.696, and accuracy of 0.654. The ADC + Cho/NAA model for *ATRX* prediction achieved a mean AUC of 0.865 ± 0.124 (95% CI: 0.780–0.953). The pooled confusion matrix showed 28 true positives, 40 true negatives, 4 false positives, and 9 false negatives, yielding a sensitivity of 0.757, specificity of 0.909, and accuracy of 0.840. The corresponding F1-scores were 0.672 and 0.812, respectively. All predictors remained statistically significant across all five folds (*p* < 0.05). The fold-specific AUCs and bootstrap AUC distributions for the *P53* and *ATRX* prediction models are shown in Figure 6 and Figure 7, respectively. Confusion matrices from the 5-fold cross-validation are shown in Figure S3.

## 4. Discussion

Our results indicate that ADC values and the Cho/NAA ratio were independent predictors of *ATRX* mutation status in *IDH*-mutant gliomas, with a combined model achieving an apparent AUC of 0.863. Furthermore, the Cho/Cr ratio was an independent predictor of *TP53* mutation status, with a model achieving an apparent AUC of 0.741. Five-fold stratified cross-validation confirmed the generalizability of both models. The Cho/Cr model achieved a cross-validated mean AUC of 0.717 ± 0.043 (Bootstrap 95% CI: 0.616–0.814), with Cho/Cr remaining significant across all five folds. The ADC + Cho/NAA model achieved a cross-validated mean AUC of 0.865 ± 0.124 (Bootstrap 95% CI: 0.780–0.953). These cross-validated metrics were highly consistent with the apparent AUCs, indicating robust generalizability with minimal overfitting.

In glioma patients, *ATRX* mutations emerge as clinically significant markers for prognosis assessment and may offer novel therapeutic opportunities. Our study found that patients with *ATRX* wild-type tumors exhibited lower ADC values, suggesting higher tumor cellularity, which aligns with previous findings. Several groups have investigated MRI-based prediction of *ATRX* status in glioma. A study reported an accuracy of 91.67% for predicting *ATRX* loss in low-grade gliomas using T2/FLAIR, ADC, and exponential ADC [27]. Leng et al. [28] employed a Lasso regression model with fifteen radiomics features extracted from multiple MRI sequences, yielding an AUC of 0.93 on the training set and 0.84 on the validation set. The higher AUCs achieved by these radiomics-based approaches, compared with the apparent AUC of 0.863 in the present study, likely reflect the greater information captured by high-dimensional radiomics features relative to individual parameters. MRS is a non-invasive technique for probing the biochemical composition of the tumor microenvironment [29]. We also observed elevated Cho/NAA ratios in *ATRX* mutant tumors. A previous study demonstrated that the T2/FLAIR mismatch sign identified *IDH*-mutant, *ATRX*-mutant astrocytoma with 100% specificity, and that combining MRS (Cho/Cr ratio) with perfusion imaging accurately distinguished grade 2 from grade 3 tumors within this molecular subgroup [30]. Multivariable logistic regression analysis confirmed that both ADC and Cho/NAA were independent predictors of *ATRX* mutation status, with a combined apparent AUC of 0.863. Bernabéu-Sanz et al. [31] found that *ATRX* wild-type high-grade gliomas had lower ADC values than *ATRX* mutant tumors, which fits with our own ADC results. In that same study, however, Cho/Cr and NAA/Cr did not differ by *ATRX* status. The gap between their MRS findings and ours may come down to differences in the cohorts—they studied high-grade gliomas and had only seven *ATRX*-mutant cases—whereas we focused on *IDH*-mutant gliomas. In our data, Cho/NAA emerged as an independent predictor, suggesting it may carry additional value in this molecularly defined subgroup.

For *TP53*, Cho/Cr was the only independent predictor in the multivariable model, yielding an apparent AUC of 0.741, sensitivity of 72.0%, and specificity of 82.0%. The cross-validated sensitivity and specificity were 0.623 and 0.696, respectively, with a mean AUC of 0.717 ± 0.043, confirming the model’s moderate but stable discriminative ability. Others have reported links between MRS parameters and *P53* status, but the direction of these correlations has not been consistent. Rudnay et al. [32] found that in patients with stable disease, Cho/Cr and Cho/NAA showed strong negative correlations with *P53* mutation, whereas in those with tumor progression, these correlations turned positive. Li et al. [33] reported a positive correlation between Cho/Cr, Cho/NAA, and *P53* expression in glioblastoma. Our finding that higher Cho/Cr was associated with *TP53* mutation aligns more closely with the positive correlations observed in the progressive subgroup in Rudnay’s study and with Li’s results. Previous studies have employed MRS for predicting molecular subtypes in gliomas [34]. Tumor heterogeneity and variation in patient mix across studies likely contribute to the inconsistent results. Future work combining MRS with perfusion or radiomics features may help clarify these relationships.

We observed that Ve was elevated in *ATRX*-mutant relative to *ATRX*-wildtype tumors (*p* = 0.029). But for most other variables, we didn’t see a clear link with *TP53* or *ATRX* status. One reason might be that our patients were mostly WHO grade II–III, so the blood–brain barrier was still relatively intact—that could make perfusion differences harder to detect. Additionally, the use of two different scanners may have introduced technical variability. Furthermore, the sample size, particularly for the *ATRX*-loss group (*n* = 81), was modest and likely reduced statistical power. In addition, single-ROI placement on the area of maximal enhancement may not adequately capture whole-tumor perfusion heterogeneity. There is not much published work on DCE-MRI and these two molecular markers, and what exists points in different directions. Hilario et al. [35] found lower leakage and Vp values in high-grade gliomas with *ATRX* loss. Other groups, using DSC-based perfusion metrics, have also reported lower rCBV and lower relative peak height in *ATRX*-mutant tumors. A possible explanation is that *ATRX* mutation alters interstitial space or the tumor microenvironment. Our other DCE data did not show these patterns. This could partly reflect differences in acquisition and patient selection—Hilario’s study targeted high-grade gliomas, whereas our cohort was limited to *IDH*-mutant gliomas.

The retrospective, single-center nature may introduce selection bias and limit the generalizability of the results. The use of different statistical methods (e.g., univariable logistic regression versus chi-square tests, Student’s t-tests, and Mann–Whitney U tests) for different variables could affect the comparability and robustness of the findings if not pre-specified. For instance, the choice between parametric and non-parametric tests hinges on data distribution, and mixing them without explicit justification may raise concerns. A unified, pre-specified statistical plan would strengthen future analyses.

This study has several limitations. First, its retrospective design and reliance on a relatively small single-institution dataset may affect generalizability. In particular, the *ATRX* subgroup was small (*n* = 81), which may have limited the statistical power to detect group differences and precluded more detailed subgroup analyses. The 5-fold cross-validation mitigated this limitation by maximizing data utilization, but external validation in larger multi-center cohorts remains essential. To address this, future work will incorporate larger, multi-center cohorts. Second, a limitation of this study is the use of single-slice 2D ROIs, which may not capture the full heterogeneity of perfusion and diffusion parameters across the entire tumor. While this method is widely used in clinical practice and supported by previous studies, whole-tumor 3D volumetric analysis in future work could offer additional value. In subsequent studies, we plan to implement advanced modeling approaches to more precisely evaluate the predictive potential of DCE-MRI parameters. Third, a technical limitation pertains to the MRI protocol: on one of the scanners (Philips Ingenia CX), different T1-weighted sequences were used before and after contrast administration. Specifically, a 2D spin-echo (SE) sequence was used pre-contrast, whereas a 3D gradient-echo sequence was used post-contrast. Although this is common clinical practice to optimize scan time and contrast resolution, the inherent differences in contrast mechanisms and spatial resolution between these sequences could introduce variability in quantitative assessments of enhancement patterns. Fourth, our study only included *IDH*-mutant gliomas. *IDH*-wildtype tumors were not evaluated. Given the distinct molecular pathogenesis of *IDH*-wildtype gliomas our findings should not be generalized to that population without independent validation. Fifth, as noted in the preceding paragraph, the use of varied statistical methods without a pre-specified plan could affect the comparability of findings. A unified, pre-specified statistical plan would strengthen future analyses. Finally, while internal validation via cross-validation supports the robustness of our models, it does not replace external validation. The complete separation observed in some *ATRX* cross-validation folds suggests that the ADC + Cho/NAA model may have overestimated discriminative ability in the current cohort, and its performance should be evaluated in independent datasets.

## 5. Conclusions

Morphological MRI, MRS, DWI, and DCE-PWI demonstrated predictive potential for *ATRX* mutation status and *TP53* gene status in *IDH*-mutant gliomas. These results support the potential role of multimodal MRI in enabling non-invasive preoperative biomarker assessment. Further prospective validation in larger, multicenter cohorts is needed to establish reliable clinical thresholds.

## Figures and Tables

**Table 1:** Comparative Analysis of Clinical Characteristics and MRI Parameters of *TP53* status in *IDH*-Mutant Gliomas.

Variables	*TP53*		*p* *
	−(*n* = 46)	+(*n* = 60)	
Gender, *n*			0.117
Male	27	26	
Female	19	34	
Age	45.609 ± 14.255	46.117 ± 13.664	0.854
Degree cystic, *n*			0.222
Proportion of cystic component ≤ 25%	37	42	
Proportion of cystic component > 25%	9	18	
lesion number, *n*			0.275
Single	44	54	
Multiple	7	2	
degree_enhancement, *n*			0.018
Proportion of enhancing tumor ≤ 33%	32	28	
Proportion of enhancing tumor > 33%	14	32	
TIC_classification, *n*			
Type I	22	28	0.672
Type II	7	13	
Type III	17	19	
NAA/Cr	0.790 [0.540, 1.080]	0.650 [0.410, 0.990]	0.165
Cho/Cr	1.690 [1.350, 2.210]	2.460 [1.700, 3.620]	<0.001
Cho/NAA	2.268 [1.835, 3.517]	4.333 [2.000, 6.980]	0.005
ADC, mm^2^/s	1121.667 [973.000, 1297.333]	1156.000 [952.000, 1397.667]	0.586
Ktrans, min^−1^	58.000 [29.000, 90.000]	67.000 [36.000, 94.000]	0.505
Kep, min^−1^	799.033 [329.400, 1391.000]	485.697 [279.333, 1118.063]	0.078
Ve	18.500 [7.230, 190.900]	73.635 [12.000, 195.537]	0.077
Vp mL/100 g	2.222 [1.193, 6.239]	3.980 [1.580, 7.130]	0.195
iAUC, min·mmol/L	13.443 [2.645, 28.000]	4.608 [2.132, 16.925]	0.367

Data are expressed as the mean ± standard deviation for normally distributed variables or median [interquartile range] for non-normally distributed variables. * Categorical variables were analyzed using the χ^2^ test. Continuous variables were compared using either Student’s *t*-test or the Mann–Whitney U test, as appropriate based on distributional assumptions. −, absence of *TP53* mutation; +, presence of *TP53* mutation.

**Table 2:** Comparative Analysis of Clinical Characteristics and MRI Parameters of *ATRX* status in *IDH*-Mutant Gliomas.

Variables	*ATRX*		
	−(*n* = 44)	+(*n* = 37)	*p* *
Gender, *n*			0.504
Male	21	16	
Female	23	21	
Age	45.561 ± 15.163	45.532 ± 13.493	0.992
Degree cystic, *n*			0.89
Proportion of cystic component ≤ 25%	19	16	
Proportion of cystic component > 25%	4	3	
lesion number, *n*			0.525
Single	40	35	
Multiple	4	2	
degree_enhancement, *n*			0.784
Proportion of enhancing tumor ≤ 33%	36	28	
Proportion of enhancing tumor > 33%	8	9	
TIC_classification, *n*			
Type I	21	19	0.544
Type II	9	7	
Type III	14	11	
NAA/Cr	0.840 [0.703, 1.062]	0.501 [0.310, 0.780]	0.078
Cho/Cr	1.870 [1.560, 2.240]	2.870 [2.040, 4.350]	0.077
Cho/NAA	2.296 [1.834, 2.638]	4.628 [2.377, 7.980]	0.037
ADC, mm^2^/s	1151.512 ± 191.693	1409.821 ± 337.564	0.012
Ktrans, min^−1^	56.930 ± 32.289	63.592 ± 38.263	0.407
Kep, min^−1^	714.300 [300.780, 1035.617]	594.747 [245.533, 1298.310]	0.836
Ve	30.627 [4.650, 113.900]	57.160 [13.087, 501.450]	0.029
Vp, mL/100 g	2.470 [0.868, 7.957]	2.809 [1.668, 6.726]	0.751
iAUC, min·mmol/L	5.358 [1.910, 10.620]	9.200 [3.843, 22.200]	0.426

Data are expressed as the mean ± standard deviation for normally distributed variables or median (interquartile range) for non-normally distributed variables. * Categorical variables were analyzed using the χ^2^ test. Continuous variables were compared using either Student’s *t*-test or the Mann–Whitney U test, as appropriate based on distributional assumptions. −, absence of *ATRX* mutation; +, presence of *ATRX* mutation.

**Table 3:** Identifying Predictors of *TP53* gene status in *IDH*-Mutant Gliomas using logistic regression analysis.

Factors	Univariable		Multivariable	
	OR (95% CI)	*p*	OR (95% CI)	*p*
Gender	1.858 [0.854, 4.045]	0.118	-	-
Age *	1.003 [0.975, 1.031]	0.852	-	-
Lesion number ^†^	2.444 [0.470, 12.716]	0.288	-	-
Cystic degree ^†^	1.762 [0.706, 4.395]	0.225	-	-
Enhancement degree ^†^	2.612 [1.165, 5.856]	0.020		
ADC	1.001 [0.999, 1.002]	0.373	-	-
TIC type ^†^				
Type I				
Type II	1.459 [0.498, 4.276]	0.491	-	-
Type III	0.878 [0.498, 4.276]	0.767	-	-
Cho/NAA	1.229 [1.061, 1.423]	0.006	1.095	0.28
Cho/Cr	2.582 [1.485, 4.488]	0.001	2.037	0.021
NAA/Cr	0.989 [0.463, 2.112]	0.977		
K^trans^ *	1.004 [0.993, 1.015]	0.493	-	-
K_ep_	1 [0.999, 1.002]	0.049	-	0.517
V_e_ *	1.002 [0.999, 1.005]	0.111	0.996	
V_p_ *	0.998 [0.989, 1.006]	0.589	-	-
iAUC *	0.991 [0.97, 1.012]	0.390	-	-

* For continuous variables, odds ratios (ORs) represent the change in odds per one-unit increase. ^†^ For categorical variables, ORs are presented relative to the reference category. Abbreviations: CI, confidence interval; ADC, apparent diffusion coefficient; TIC, time–intensity curve; iAUC, initial area under the curve.

**Table 4:** Identifying Predictors of *ATRX* status in *IDH*-Mutant Gliomas using logistic regression analysis.

Factors	Univariate		Multivariate	
	OR (95% CI)	*p*	OR (95% CI)	*p*
Gender	1.199 [0.514, 2.796]	0.676	-	-
Age *	1.001 [0.970, 1.031]	0.992	-	-
Lesion number ^†^	0.571 [0.098, 3.337]	0.535	-	-
Cystic degree ^†^	0.871 [0.271, 2.795]	0.818	-	-
Enhancement degree ^†^	1.488 [0.624, 3.551]	0.373		
ADC	1.004 [1.002, 1.006]	0.018	1.005	0.017
TIC type ^†^				
Type I				
Type II	0.894 [0.299, 2.672]	0.841	-	-
Type III	0.861 [0.324, 2.287]	0.764	-	-
Cho/NAA	2.521 [1.490, 4.267]	0.024	3.073	0.023
Cho/Cr	3.746 [1.858, 7.551]	0.057		
NAA/Cr	0.237 [0.069, 0.821]	0.063		
K^trans^ *	1.006 [0.993, 1.019]	0.395	-	-
K_ep_	1 [0.999, 1.001]	0.695	-	-
V_e_ *	1.003 [1.001, 1.006]	0.008		
V_p_ *	0.952 [0.869, 1.042]	0.288	-	-
iAUC *	0.992 [0.955, 1.030]	0.675	-	-

* For continuous variables, odds ratios (ORs) represent the change in odds per one-unit increase. ^†^ For categorical variables, ORs are presented relative to the reference category. Abbreviations: CI, confidence interval; ADC, apparent diffusion coefficient; TIC, time–intensity curve; iAUC, initial area under the concentration–time curve.

## Data Availability

The data that support the findings of this study are available from the corresponding author, Xin Zhang, on reasonable request.

## Supplementary Materials

The following supporting information can be downloaded at: https://www.mdpi.com/article/10.3390/diagnostics16142174/s1, Figure S1: The overview of the patient enrollment; Figure S2: Representative ROI placements across different MRI image sequences; Figure S3: Confusion matrices of the prediction models from 5-fold cross-validation; Table S1: The parameters of MRI; Table S2: Interobserver agreement for quantitative parameter analysis. DCE-MRI Acquisition and Processin.

## Abbreviations

The following abbreviations are used in this manuscript:

ADC	Apparent Diffusion Coefficient
*ATRX*	X-linked alpha-thalassemia intellectual disability syndrome
AUC	Area Under the Curve
CE-T1WI	Contrast-Enhanced T1-Weighted Imaging
Cho	Choline
CNS	Central Nervous System
Cr	Creatinine
DCE-PWI	Dynamic Contrast-Enhanced Perfusion-Weighted Imaging
DWI	Diffusion-Weighted Imaging
EES	Extravascular Extracellular Space
FLAIR	Fluid-Attenuated Inversion Recovery
iAUC	initial Area Under the concentration––time curve
*IDH*	Isocitrate Dehydrogenase
Kep	rate constant between EES and plasma
Ktrans	transfer constant from plasma into EES
MRI	Magnetic Resonance Imaging
MRS	Magnetic Resonance Spectroscopy
NAA	N-Acetylaspartate
OR	Odds Ratio
ROC	Receiver Operating Characteristic
ROI	Region of Interest
SE	Spin-Echo
TIC	Time–Intensity Curve
T1WI	T1-Weighted Imaging
T2WI	T2-Weighted Imaging
Ve	volume fraction of EES
VIF	Variance Inflation Factor
Vp	volume fraction of blood plasma
WHO	World Health Organization

## References

[1] Louis D.N., Perry A., Wesseling P., Brat D.J., Cree I.A., Figarella-Branger D., Hawkins C., Ng H.K., Pfister S.M., Reifenberger G. (2021). The 2021 WHO Classification of Tumors of the Central Nervous System: A summary. Neuro Oncol..

[2] Kurokawa R., Kurokawa M., Baba A., Ota Y., Pinarbasi E., Camelo-Piragua S., Capizzano A.A., Liao E., Srinivasan A., Moritani T. (2022). Major Changes in 2021 World Health Organization Classification of Central Nervous System Tumors. Radiographics.

[3] Horbinski C., Berger T., Packer R.J., Wen P.Y. (2022). Clinical implications of the 2021 edition of the WHO classification of central nervous system tumours. Nat. Rev. Neurol..

[4] Schaff L.R., Mellinghoff I.K. (2023). Glioblastoma and Other Primary Brain Malignancies in Adults: A Review. JAMA.

[5] Li Y., Liu X., Qian Z., Sun Z., Xu K., Wang K., Fan X., Zhang Z., Li S., Wang Y. (2018). Genotype prediction of *ATRX* mutation in lower-grade gliomas using an MRI radiomics signature. Eur. Radiol..

[6] Wiestler B., Capper D., Holland-Letz T., Korshunov A., von Deimling A., Pfister S.M., Platten M., Weller M., Wick W. (2013). *ATRX* loss refines the classification of anaplastic gliomas and identifies a subgroup of *IDH* mutant astrocytic tumors with better prognosis. Acta Neuropathol..

[7] Cai J., Yang P., Zhang C., Zhang W., Liu Y., Bao Z., Liu X., Du W., Wang H., Jiang T. (2014). *ATRX* mRNA expression combined with *IDH*1/2 mutational status and Ki-67 expression refines the molecular classification of astrocytic tumors: Evidence from the whole transcriptome sequencing of 169 samples. Oncotarget.

[8] Haase S., Garcia-Fabiani M.B., Carney S., Altshuler D., Nunez F.J., Mendez F.M., Nunez F., Lowenstein P.R., Castro M.G. (2018). Mutant *ATRX*: Uncovering a new therapeutic target for glioma. Expert Opin. Ther. Targets.

[9] Paunu N., Syrjakoski K., Sankila R., Simola K.O., Helen P., Niemela M., Matikainen M., Isola J., Haapasalo H. (2001). Analysis of *P53* tumor suppressor gene in families with multiple glioma patients. J. Neurooncol..

[10] Brat D.J., Verhaak R.G.W., Aldape K.D., Yung W.K.A., Salama S.R., Cooper L.A., Rheinbay E., Miller C.R., Vitucci M., Cancer Genome Atlas Research Network (2015). Comprehensive, integrative genomic analysis of diffuse lower-grade gliomas. N. Engl. J. Med..

[11] Shiraishi S., Tada K., Nakamura H., Makino K., Kochi M., Saya H., Kuratsu J., Ushio Y. (2002). Influence of *P53* mutations on prognosis of patients with glioblastoma. Cancer.

[12] Lang F.F., Bruner J.M., Fuller G.N., Aldape K., Prados M.D., Chang S., Berger M.S., McDermott M.W., Kunwar S.M., Junck L.R. (2003). Phase I trial of adenovirus-mediated *P53* gene therapy for recurrent glioma: Biological and clinical results. J. Clin. Oncol..

[13] Jin Y., Xiao W., Song T., Feng G., Dai Z. (2016). Expression and Prognostic Significance of *P53* in Glioma Patients: A Meta-analysis. Neurochem. Res..

[14] Chaumeil M.M., Lupo J.M., Ronen S.M. (2015). Magnetic Resonance (MR) Metabolic Imaging in Glioma. Brain Pathol..

[15] Hyare H., Rice L., Thust S., Nachev P., Jha A., Milic M., Brandner S., Rees J. (2019). Modelling MR and clinical features in grade II/III astrocytomas to predict *IDH* mutation status. Eur. J. Radiol..

[16] Broen M.P.G., Smits M., Wijnenga M.M.J., Dubbink H.J., Anten M.H.M.E., Schijns O.E.M.G., Beckervordersandforth J., Postma A.A., van den Bent M.J. (2018). The T2-FLAIR mismatch sign as an imaging marker for non-enhancing *IDH*-mutant, 1p/19q-intact lower-grade glioma: A validation study. Neuro Oncol..

[17] Zhang J., Peng H., Wang Y.L., Xiao H.F., Cui Y.Y., Bian X.B., Zhang D.K., Ma L. (2021). Predictive Role of the Apparent Diffusion Coefficient and MRI Morphologic Features on *IDH* Status in Patients with Diffuse Glioma: A Retrospective Cross-Sectional Study. Front. Oncol..

[18] Zhang L., Min Z., Tang M., Chen S., Lei X., Zhang X. (2017). The utility of diffusion MRI with quantitative ADC measurements for differentiating high-grade from low-grade cerebral gliomas: Evidence from a meta-analysis. J. Neurol. Sci..

[19] Zeng Q., Dong F., Shi F., Ling C., Jiang B., Zhang J. (2017). Apparent diffusion coefficient maps obtained from high b value diffusion-weighted imaging in the preoperative evaluation of gliomas at 3T: Comparison with standard b value diffusion-weighted imaging. Eur. Radiol..

[20] Larsson C., Kleppesto M., Grothe I., Vardal J., Bjornerud A., Emblem K.E. (2015). T1 in high-grade glioma and the influence of different measurement strategies on parameter estimations in DCE-MRI. J. Magn. Reson. Imaging.

[21] Brendle C., Hempel J.M., Schittenhelm J., Skardelly M., Tabatabai G., Bender B., Ernemann U., Klose U. (2018). Glioma Grading and Determination of *IDH* Mutation Status and *ATRX* loss by DCE and ASL Perfusion. Clin. Neuroradiol..

[22] Fabijanska A. (2016). A novel approach for quantification of time-intensity curves in a DCE-MRI image series with an application to prostate cancer. Comput. Biol. Med..

[23] Pope W.B., Prins R.M., Albert Thomas M., Nagarajan R., Yen K.E., Bittinger M.A., Salamon N., Chou A.P., Yong W.H., Soto H. (2012). Non-invasive detection of 2-hydroxyglutarate and other metabolites in *IDH*1 mutant glioma patients using magnetic resonance spectroscopy. J. Neurooncol..

[24] Lv X.Q., Shen W.R., Guo Z., Xie X.D. (2025). Diagnostic value and efficacy of multimodal magnetic resonance imaging in differentiating radiation necrosis from tumor recurrence in glioblastomas. Acta Radiol..

[25] Crain I.D., Elias P.S., Chapple K., Scheck A.C., Karis J.P., Preul M.C. (2017). Improving the utility of 1H-MRS for the differentiation of glioma recurrence from radiation necrosis. J. Neurooncol..

[26] Yang H., Zhu Z., Long C., Niu F., Zhou J., Chen S., Ye M., Peng S., Zhang X., Chen Y. (2024). Quantitative and Qualitative Parameters of DCE-MRI Predict CDKN2A/B Homozygous Deletion in Gliomas. Acad. Radiol..

[27] Ren Y., Zhang X., Rui W., Pang H., Qiu T., Wang J., Xie Q., Jin T., Zhang H., Chen H. (2019). Noninvasive Prediction of *IDH*1 Mutation and *ATRX* Expression Loss in Low-Grade Gliomas Using Multiparametric MR Radiomic Features. J. Magn. Reson. Imaging.

[28] Meng L., Zhang R., Fa L., Zhang L., Wang L., Shao G. (2022). *ATRX* status in patients with gliomas: Radiomics analysis. Medicine.

[29] Bahrami N., Hartman S.J., Chang Y.H., Delfanti R., White N.S., Karunamuni R., Seibert T.M., Dale A.M., Hattangadi-Gluth J.A., Piccioni D. (2018). Molecular classification of patients with grade II/III glioma using quantitative MRI characteristics. J. Neurooncol..

[30] Sawlani V., Jen J.P., Patel M., Jain M., Haq H., Ughratdar I., Wykes V., Nagaraju S., Watts C., Pohl U. (2024). Multiparametric MRI and T2/FLAIR mismatch complements the World Health Organization 2021 classification for the diagnosis of *IDH*-mutant 1p/19q non-co-deleted/*ATRX*-mutant astrocytoma. Clin. Radiol..

[31] Bernabéu-Sanz Á., Fuentes-Baile M., Alenda C. (2021). Main genetic differences in high-grade gliomas may present different MR imaging and MR spectroscopy correlates. Eur. Radiol..

[32] Rudnay M., Waczulikova I., Bullova A., Rjaskova G., Chorvath M., Jezberova M., Lehotska V. (2021). Magnetic resonance spectroscopy—Its added value in brain glioma multiparametric assessment. Bratisl. Lek. Listy.

[33] Li Y., Ji F., Jiang Y., Zhao T., Xu C. (2018). Correlation analysis of expressions of PTEN and *P53* with the value obtained by magnetic resonance spectroscopy and apparent diffusion coefficient in the tumor and the tumor-adjacent area in magnetic resonance imaging for glioblastoma. J. BUON.

[34] Ozturk-Isik E., Cengiz S., Ozcan A., Yakicier C., Ersen Danyeli A., Pamir M.N., Ozduman K., Dincer A. (2020). Identification of *IDH* and TERTp mutation status using 1H-MRS in 112 hemispheric diffuse gliomas. J. Magn. Reson. Imaging.

[35] Hilario A., Hernandez-Lain A., Sepulveda J.M., Lagares A., Perez-Nuñez A., Ramos A. (2019). Perfusion MRI grading diffuse gliomas: Impact of permeability parameters on molecular biomarkers and survival. Neurocirugia.

