# Supplementary material for: Noninvasive Prediction of TP53 Gene Status and ATRX Gene Status in IDH-Mutant Glioma Using Multimodal MRI: Incorporating Morphological, Spectroscopic, Diffusion, and Perfusion Imaging
Source: Diagnostics (Basel). 2026 Jul 12;16(14):2174. doi: 10.3390/diagnostics16142174 (PMC13408992; doi:10.3390/diagnostics16142174)
Supplement: Supplementary file 1 [file diagnostics-16-02174-s001.zip › diagnostics-4407628-Supplementary.pdf]

Supplementary Materials

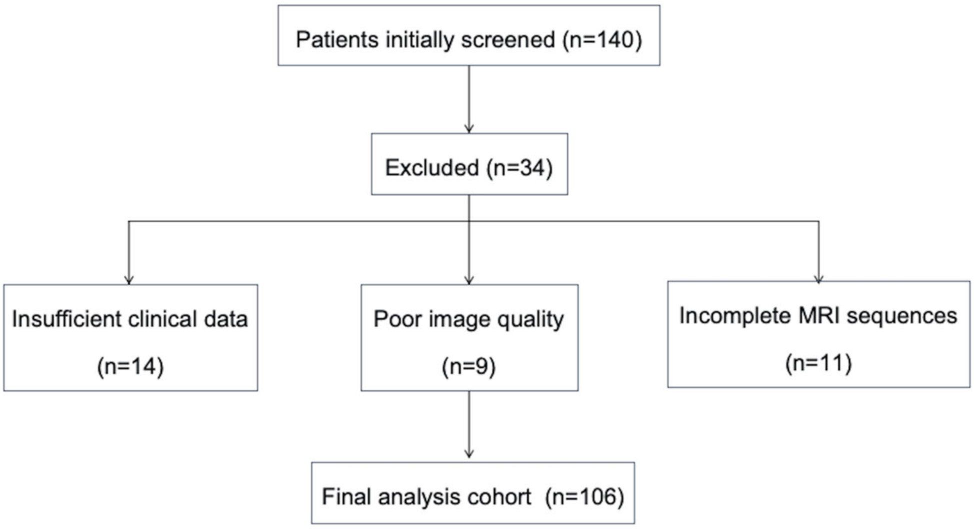

Figure S1. The overview of the patient enrollment. A total of 140 patients with pathologically confirmed WHO grade 2–4 gliomas were initially screened. After excluding 34 patients due to insufficient clinical data (n=14), poor image quality (n=9), or incomplete MRI sequences (n=11), 106 patients were included in the final analysis.

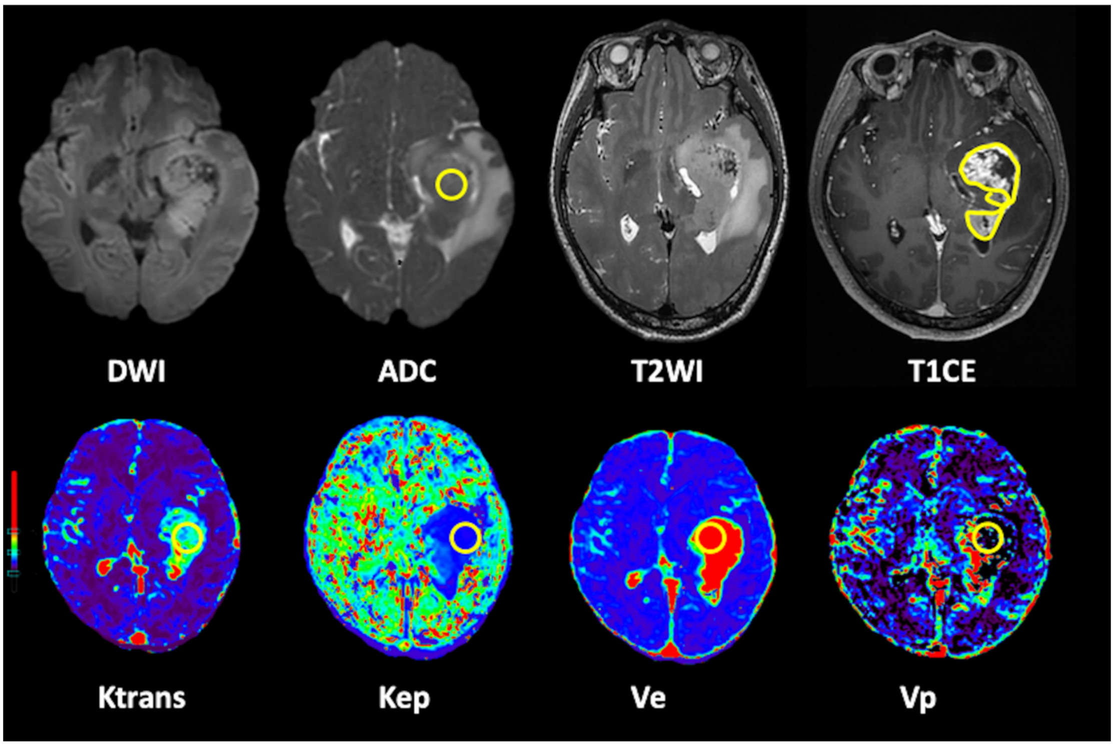

Figure S2. Representative ROI placements across different MRI image sequences. Regions of interest (ROIs) are delineated in yellow. ROIs are shown on each imaging sequence or parameter map, including DWI, ADC, T2WI, T1CE, Ktrans, Kep, Ve. ROIs were consistently placed on the solid enhancing portion of the tumor. A circular region of interest (ROI) measuring 10-15 mm^2 was applied to the ADC maps and all DCE parameter maps.

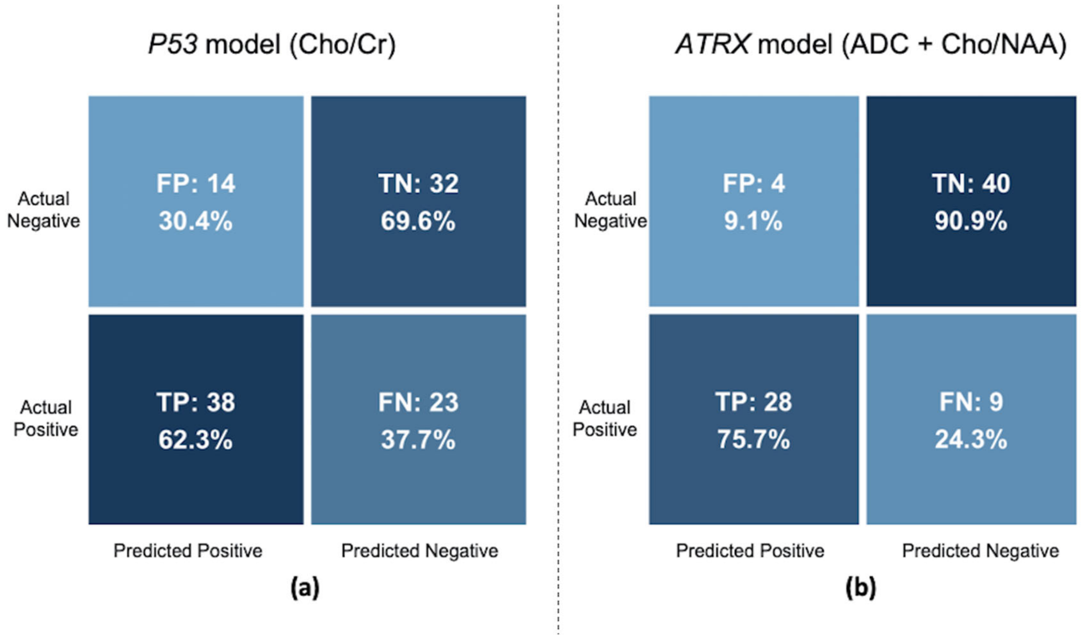

Figure S3. Confusion matrices of the prediction models from 5-fold cross-validation. (a) P53 model (Cho/Cr): 32 true negatives, 14 false positives, 23 false negatives, 38 true positives. (b) ATRX model (ADC + ChoNAA): 40 true negatives, 4 false positives, 9 false negatives, 28 true positives. The color intensity reflects the count in each cell, with darker blue indicating higher values. TP, true positive; TN, true negative; FP, false positive; FN, false negative.

Table S1. The parameters of MRI

Scanning machine	sequence	layer thickness /mm	voxel size/mm3	FOV/mm 2	TR/ms	TE/ms	flip angle/ °	time resolution /s	total acquisition time/s
uMR790 (3.0T)	T1WI	1	1×1×1	256×232	7.9	3.1	10	—	74
	T2WI	1	1×1×1	256×232	220	606.4	90	—	93
	3D-T1CE	1	1×1×1	256×232	7.9	3.1	10	—	74
	DWI	5	1.6×1.6×5.0	230×230	3598	108.1	90	—	345
	DCE	5	1.5×1.5×5.0	240×220	3.47	1.9	13	4	369
	MRS	—	15×15×20	—	2000	144	90	—	259
Philips Ingenia CX(3.0 T)	T1WI	5	0.90×1.12×5.00	230×183	250	2.3	75	—	70
	T2WI	1	1×1×1	240×240	3000	280	90	—	67
	3D-T1CE	1	1×1×1	240×240	6.6	3	8	—	64
	DWI	5	1.6×2×5.0	230×230	2988	90	90	—	380
	DCE	5	1×1×5	220×189	3.8	1.83	15	4	392
	MRS	—	20×20×20	—	2000	144	90	—	268

Table S2. Interobserver agreement for quantitative parameter analysis

Variables	Interobserver agreement	
	Cohen kappa index	95% confidence interval
ADC	0.880	0.875-0.886
K^{trans}	0.897	0.895-0.899
K_{ep}	0.906	0.886-0.926
V_e	0.898	0.896-0.899
V_p	0.854	0.851-0.858
iAUC	0.895	0.891-0.898

DCE-MRI Acquisition and Processing

DCE-MRI was performed on two 3.0T scanners: a United Imaging uMR 790 and a Philips Ingenia CX. Both used a 3D T1-weighted gradient-echo sequence. For the United Imaging system, the parameters were: slice thickness 5 mm, voxel size 1.5 × 1.5 × 5.0 mm^3, FOV 240 × 220 mm^2, TR/TE 3.47/1.9 ms, flip angle 13°, temporal resolution 4 s, and total acquisition time 369 s (92 phases). For the Philips system: slice thickness 5 mm, voxel size 1 × 1 × 5 mm^3, FOV 220 × 189 mm^2, TR/TE 3.8/1.83 ms, flip angle 15°, temporal resolution 4 s, total acquisition time 392 s (98 phases).

Gadodiamide (Omniscan, GE Healthcare) was injected at 0.1 mmol/kg body weight via an antecubital vein at 3 mL/s, followed by a 20 mL saline flush at the same rate. Injection started after five baseline phases.

AIF Determination

AIF was determined for each patient using a semi-automated method with four steps.

First, candidate voxels were automatically selected from the middle cerebral artery (M1 segment) or supraclinoid internal carotid artery on the source DCE images. Selection criteria were: time to peak < 15 s, peak signal > 3 times the mean baseline signal (first five phases), and full-width at half-maximum < 10 s.

Second, a k-means clustering algorithm (k = 3) was applied to these voxels based on their enhancement kinetics (time to peak, peak enhancement, area under the curve). The cluster with the earliest time to peak and highest peak-to-baseline ratio was kept. This was done using the scanner's native software (uWS-MR post-processing workstation for United Imaging, IntelliSpace Portal for Philips).

Third, a neuroradiologist with 10 years of experience reviewed all candidate voxels from the selected cluster (usually 3–10 voxels). Voxels were removed if they showed partial volume artifact, flow void, vessel wall contamination, or motion distortion. After review, 3 to 5 voxels were kept per patient.

Fourth, for each kept voxel, the signal-time curve was converted to a concentration-time curve using:

$$C(t) = -\frac{1}{r_1 \cdot TE} \cdot \ln\left(\frac{S(t)}{S_0}\right)$$

where r_1 = 4.3 L/mmol/s for gadodiamide at 3.0T, TE is the echo time, S(t) is the signal at time t, and S_0 is the mean baseline signal. A gamma-variate function was fitted to each curve to remove recirculation and smooth noise. The final patient-specific AIF was the average of the fitted curves from the 3–5 kept voxels. If an AIF had a peak concentration < 1.5 mM or showed a visible second peak, the patient was excluded from DCE analysis.

Pharmacokinetic Modeling

The extended Tofts model was applied voxel-by-voxel to generate Ktrans, Kep, and Ve maps:

$$C_t(t) = K^{trans} \int_0^t C_p(\tau) \cdot e^{-k_{ep}(t-\tau)} d\tau + v_p C_p(t)$$

C_t(t) is tissue concentration at time t, C_p(t) is plasma concentration from the patient-specific AIF, Ktrans is the transfer constant from plasma to the extravascular extracellular space, and kep = Ktrans/Ve. Fitting was done with the commercial software (uWS-MR or IntelliSpace Portal). For each patient, the same AIF was used for all voxels and all parameters.

DCE Exclusion Criteria

Patients were excluded from DCE analysis if: (a) fewer than 3 valid AIF voxels were identified, (b) AIF peak concentration < 1.5 mM, (c) significant motion artifacts on DCE source images (visual rating ≥ 2 on a 4-point scale), or (d) incomplete DCE series.
